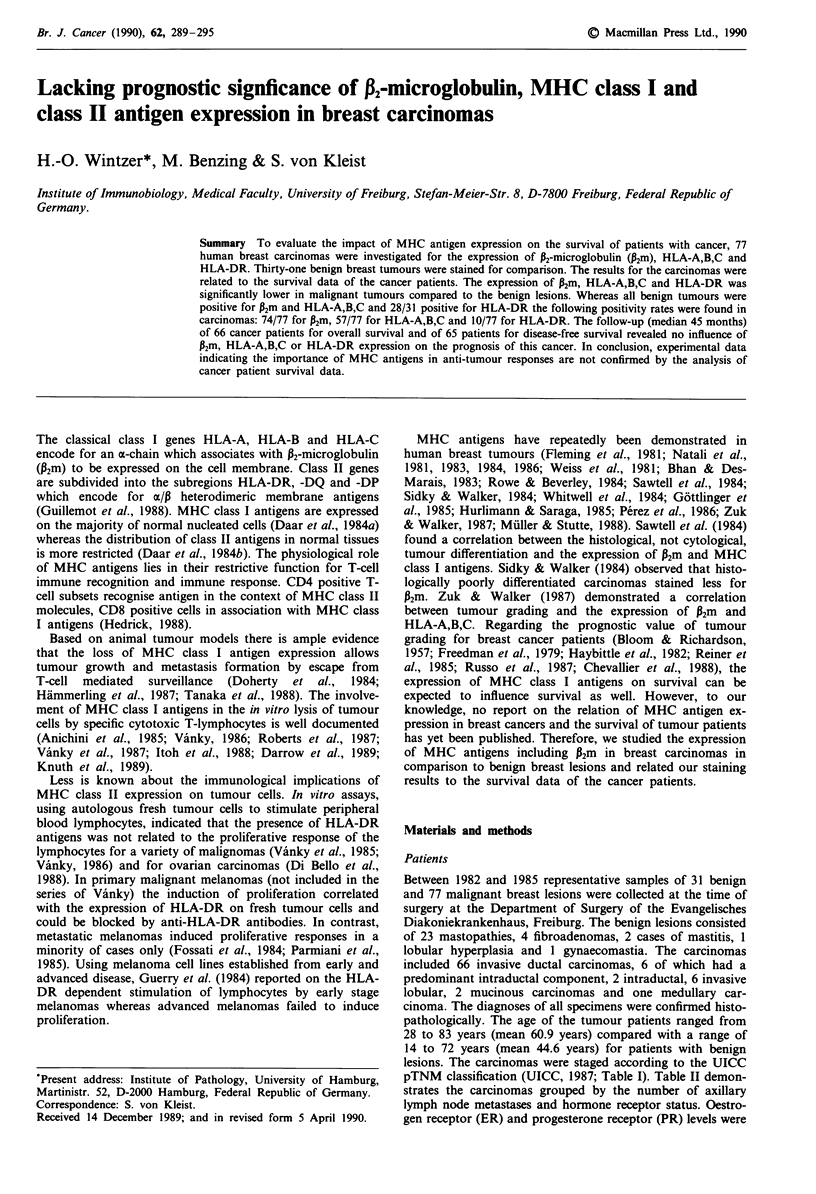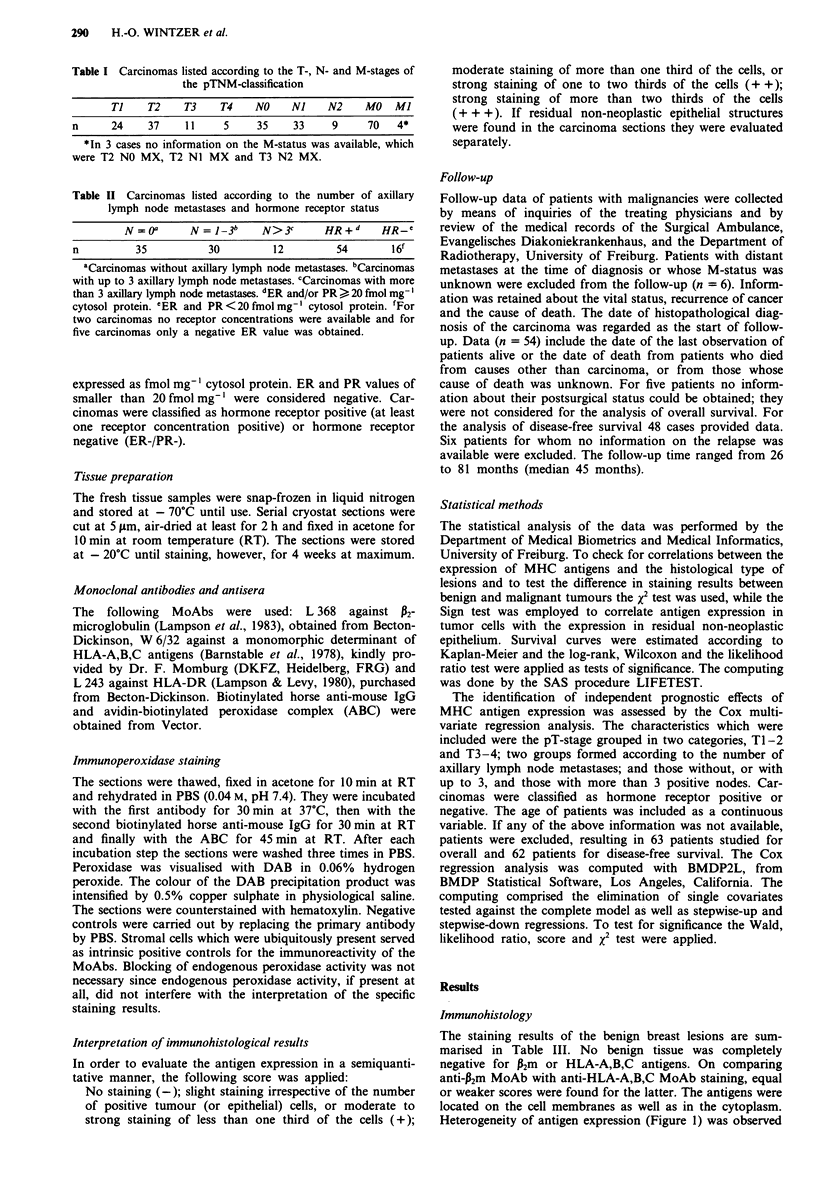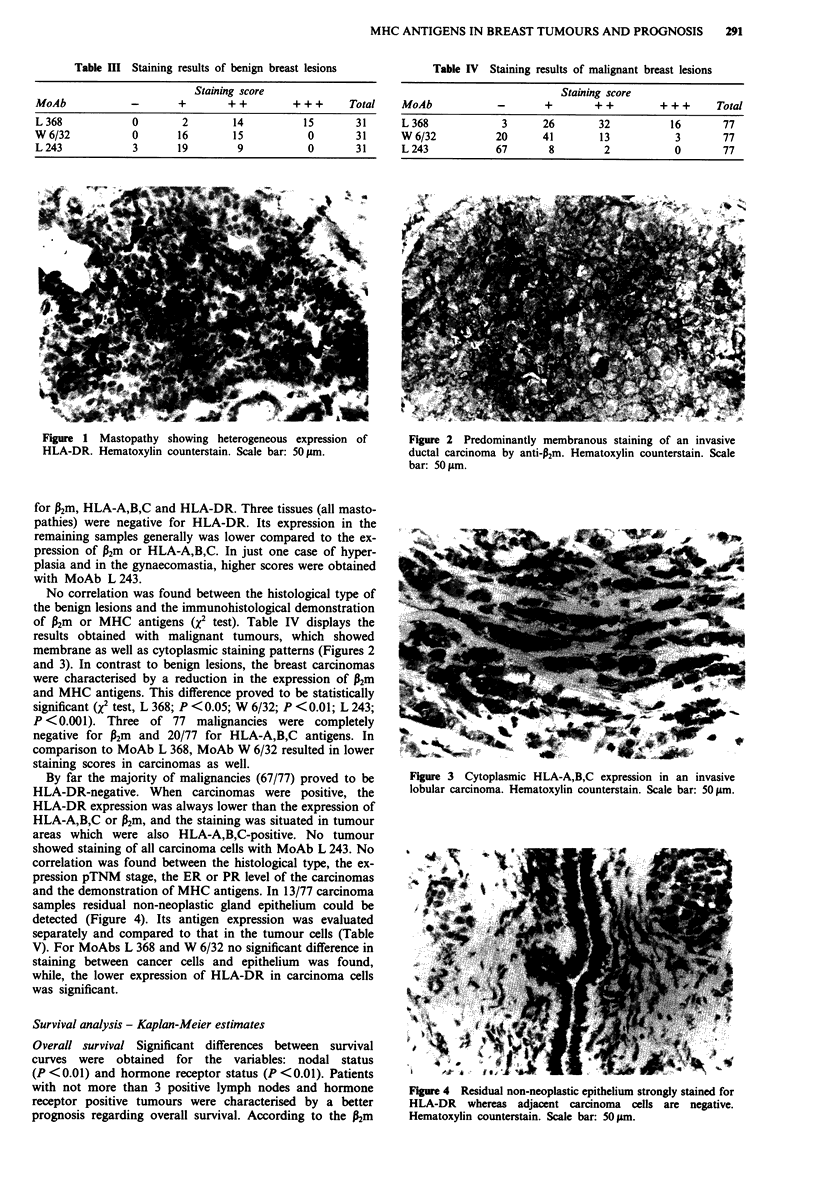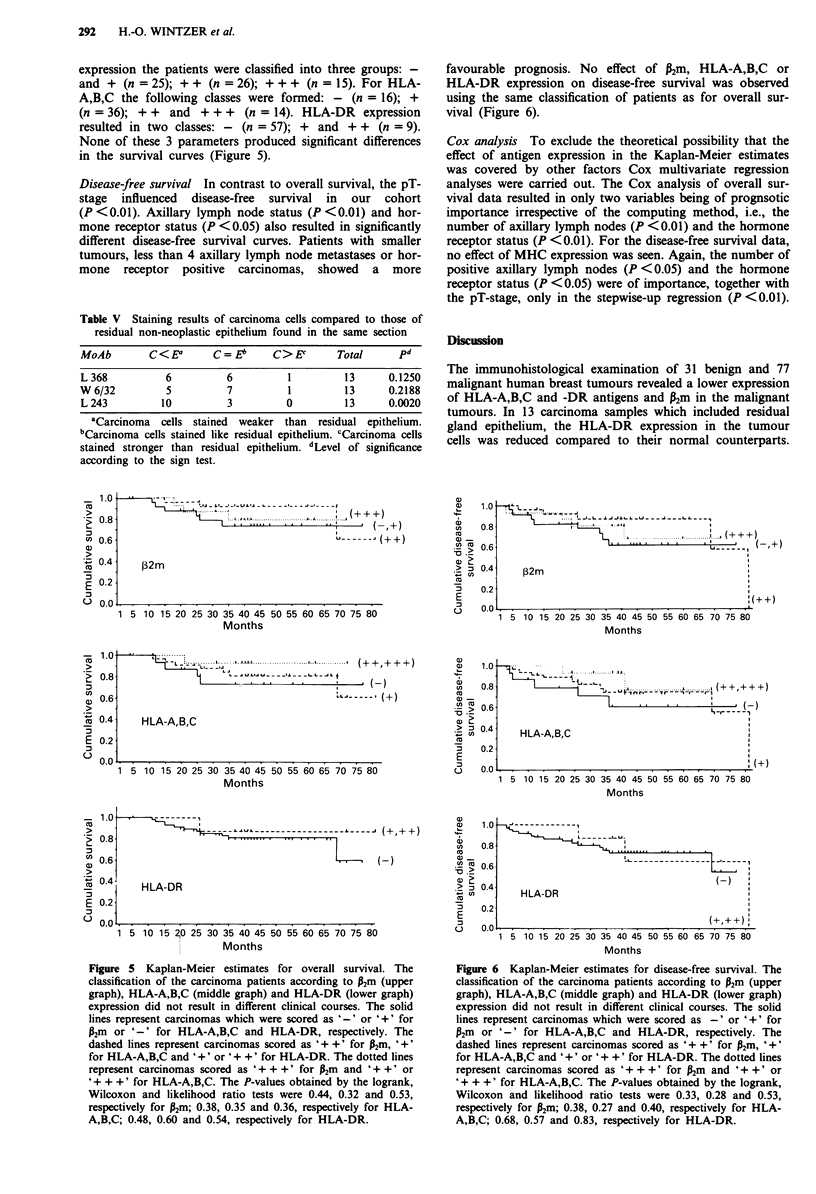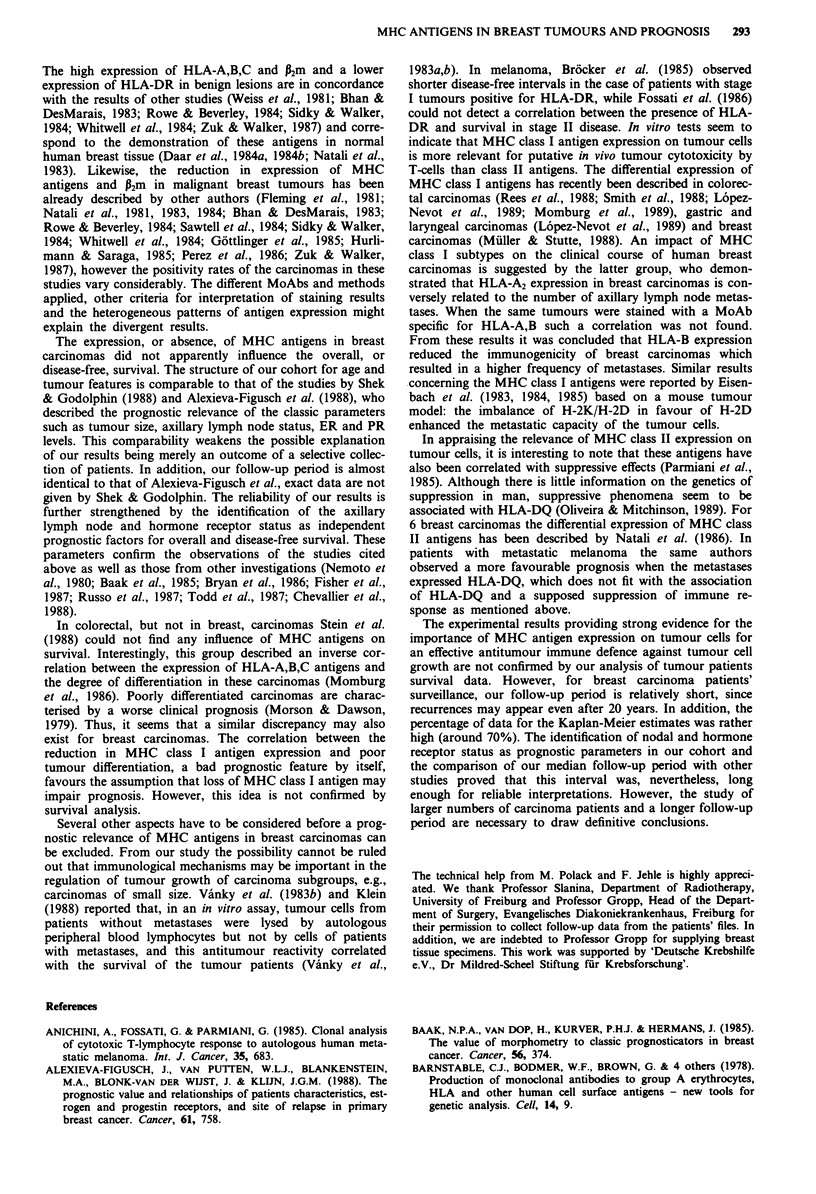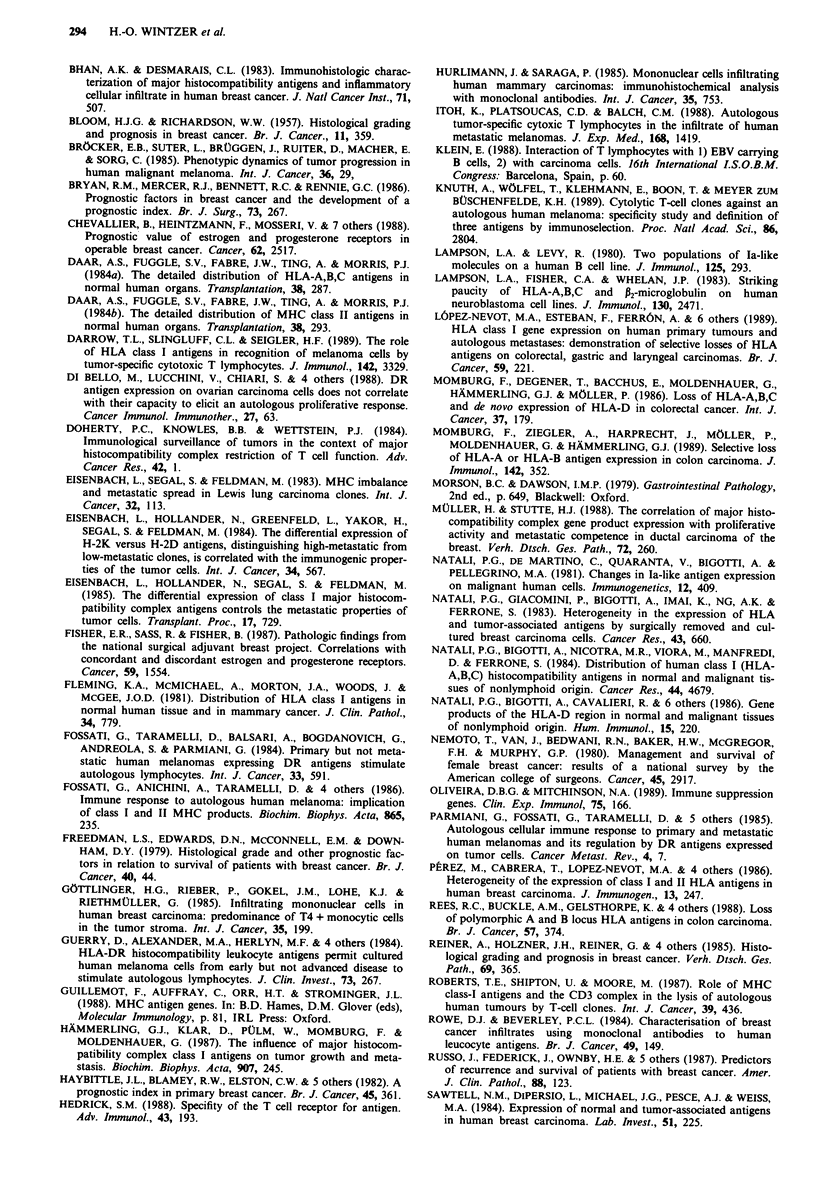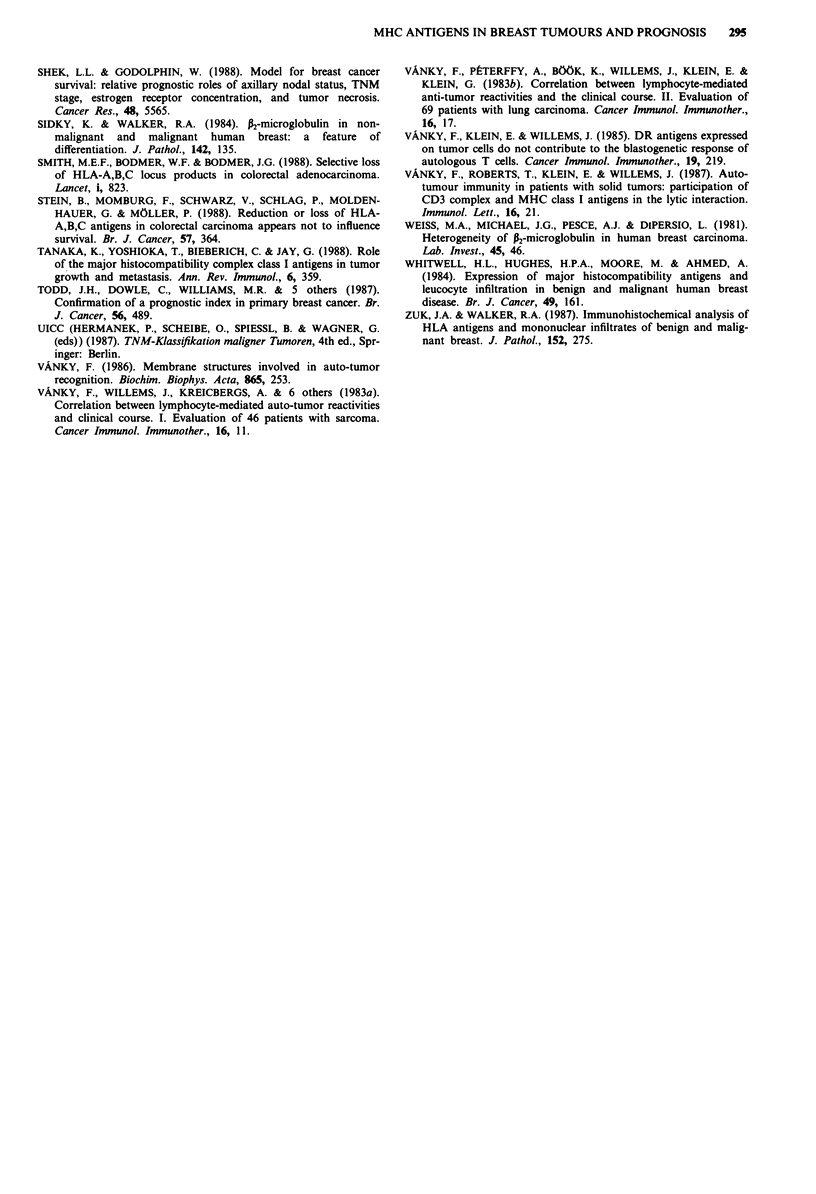# Lacking prognostic significance of beta 2-microglobulin, MHC class I and class II antigen expression in breast carcinomas.

**DOI:** 10.1038/bjc.1990.280

**Published:** 1990-08

**Authors:** H. O. Wintzer, M. Benzing, S. von Kleist

**Affiliations:** Institute of Immunobiology, Medical Faculty, University of Freiburg, Federal Republic of Germany.

## Abstract

**Images:**


Br. J. Cancer (1990), 62, 289–295 © Macmillan Press Ltd., 1990

### Lacking prognostic signficance of $\beta_2$-microglobulin, MHC class I and class II antigen expression in breast carcinomas

H.-O. Wintzer*, M. Benzing & S. von Kleist

*Institute of Immunobiology, Medical Faculty, University of Freiburg, Stefan-Meier-Str. 8, D-7800 Freiburg, Federal Republic of Germany.*

**Summary** To evaluate the impact of MHC antigen expression on the survival of patients with cancer, 77 human breast carcinomas were investigated for the expression of $\beta_2$-microglobulin ($\beta_2$m), HLA-A,B,C and HLA-DR. Thirty-one benign breast tumours were stained for comparison. The results for the carcinomas were related to the survival data of the cancer patients. The expression of $\beta_2$m, HLA-A,B,C and HLA-DR was significantly lower in malignant tumours compared to the benign lesions. Whereas all benign tumours were positive for $\beta_2$m and HLA-A,B,C and 28/31 positive for HLA-DR the following positivity rates were found in carcinomas: 74/77 for $\beta_2$m, 57/77 for HLA-A,B,C and 10/77 for HLA-DR. The follow-up (median 45 months) of 66 cancer patients for overall survival and of 65 patients for disease-free survival revealed no influence of $\beta_2$m, HLA-A,B,C or HLA-DR expression on the prognosis of this cancer. In conclusion, experimental data indicating the importance of MHC antigens in anti-tumour responses are not confirmed by the analysis of cancer patient survival data.

The classical class I genes HLA-A, HLA-B and HLA-C encode for an α-chain which associates with $\beta_2$-microglobulin ($\beta_2$m) to be expressed on the cell membrane. Class II genes are subdivided into the subregions HLA-DR, -DQ and -DP which encode for α/β heterodimeric membrane antigens (Guillemot *et al.*, 1988). MHC class I antigens are expressed on the majority of normal nucleated cells (Daar *et al.*, 1984*a*) whereas the distribution of class II antigens in normal tissues is more restricted (Daar *et al.*, 1984*b*). The physiological role of MHC antigens lies in their restrictive function for T-cell immune recognition and immune response. CD4 positive T-cell subsets recognise antigen in the context of MHC class II molecules, CD8 positive cells in association with MHC class I antigens (Hedrick, 1988).

Based on animal tumour models there is ample evidence that the loss of MHC class I antigen expression allows tumour growth and metastasis formation by escape from T-cell mediated surveillance (Doherty *et al.*, 1984; Hämmerling *et al.*, 1987; Tanaka *et al.*, 1988). The involvement of MHC class I antigens in the *in vitro* lysis of tumour cells by specific cytotoxic T-lymphocytes is well documented (Anichini *et al.*, 1985; Vánky, 1986; Roberts *et al.*, 1987; Vánky *et al.*, 1987; Itoh *et al.*, 1988; Darrow *et al.*, 1989; Knuth *et al.*, 1989).

Less is known about the immunological implications of MHC class II expression on tumour cells. *In vitro* assays, using autologous fresh tumour cells to stimulate peripheral blood lymphocytes, indicated that the presence of HLA-DR antigens was not related to the proliferative response of the lymphocytes for a variety of malignomas (Vánky *et al.*, 1985; Vánky, 1986) and for ovarian carcinomas (Di Bello *et al.*, 1988). In primary malignant melanomas (not included in the series of Vánky) the induction of proliferation correlated with the expression of HLA-DR on fresh tumour cells and could be blocked by anti-HLA-DR antibodies. In contrast, metastatic melanomas induced proliferative responses in a minority of cases only (Fossati *et al.*, 1984; Parmiani *et al.*, 1985). Using melanoma cell lines established from early and advanced disease, Guerry *et al.* (1984) reported on the HLA-DR dependent stimulation of lymphocytes by early stage melanomas whereas advanced melanomas failed to induce proliferation.

*Present address: Institute of Pathology, University of Hamburg, Martinistr. 52, D-2000 Hamburg, Federal Republic of Germany.
Correspondence: S. von Kleist.
Received 14 December 1989; and in revised form 5 April 1990.

MHC antigens have repeatedly been demonstrated in human breast tumours (Fleming *et al.*, 1981; Natali *et al.*, 1981, 1983, 1984, 1986; Weiss *et al.*, 1981; Bhan & Des-Marais, 1983; Rowe & Beverley, 1984; Sawtell *et al.*, 1984; Sidky & Walker, 1984; Whitwell *et al.*, 1984; Göttlinger *et al.*, 1985; Hurlimann & Saraga, 1985; Pérez *et al.*, 1986; Zuk & Walker, 1987; Müller & Stutte, 1988). Sawtell *et al.* (1984) found a correlation between the histological, not cytological, tumour differentiation and the expression of $\beta_2$m and MHC class I antigens. Sidky & Walker (1984) observed that histologically poorly differentiated carcinomas stained less for $\beta_2$m. Zuk & Walker (1987) demonstrated a correlation between tumour grading and the expression of $\beta_2$m and HLA-A,B,C. Regarding the prognostic value of tumour grading for breast cancer patients (Bloom & Richardson, 1957; Freedman *et al.*, 1979; Haybittle *et al.*, 1982; Reiner *et al.*, 1985; Russo *et al.*, 1987; Chevallier *et al.*, 1988), the expression of MHC class I antigens on survival can be expected to influence survival as well. However, to our knowledge, no report on the relation of MHC antigen expression in breast cancers and the survival of tumour patients has yet been published. Therefore, we studied the expression of MHC antigens including $\beta_2$m in breast carcinomas in comparison to benign breast lesions and related our staining results to the survival data of the cancer patients.

### Materials and methods

### *Patients*

Between 1982 and 1985 representative samples of 31 benign and 77 malignant breast lesions were collected at the time of surgery at the Department of Surgery of the Evangelisches Diakoniekrankenhaus, Freiburg. The benign lesions consisted of 23 mastopathies, 4 fibroadenomas, 2 cases of mastitis, 1 lobular hyperplasia and 1 gynaecomastia. The carcinomas included 66 invasive ductal carcinomas, 6 of which had a predominant intraductal component, 2 intraductal, 6 invasive lobular, 2 mucinous carcinomas and one medullary carcinoma. The diagnoses of all specimens were confirmed histopathologically. The age of the tumour patients ranged from 28 to 83 years (mean 60.9 years) compared with a range of 14 to 72 years (mean 44.6 years) for patients with benign lesions. The carcinomas were staged according to the UICC pTNM classification (UICC, 1987; Table I). Table II demonstrates the carcinomas grouped by the number of axillary lymph node metastases and hormone receptor status. Oestrogen receptor (ER) and progesterone receptor (PR) levels were

**Table I** Carcinomas listed according to the T-, N- and M-stages of the pTNM-classification

| | *T1* | *T2* | *T3* | *T4* | *N0* | *N1* | *N2* | *M0* | *M1* |
|---|---|---|---|---|---|---|---|---|---|
| n | 24 | 37 | 11 | 5 | 35 | 33 | 9 | 70 | 4* |

*In 3 cases no information on the M-status was available, which were T2 N0 MX, T2 N1 MX and T3 N2 MX.

**Table II** Carcinomas listed according to the number of axillary lymph node metastases and hormone receptor status

| | *N = 0*[a] | *N = 1–3*[b] | *N>3*[c] | *HR +*[d] | *HR –*[e] |
|---|---|---|---|---|---|
| n | 35 | 30 | 12 | 54 | 16[f] |

[a]Carcinomas without axillary lymph node metastases. [b]Carcinomas with up to 3 axillary lymph node metastases. [c]Carcinomas with more than 3 axillary lymph node metastases. [d]ER and/or PR ⩾ 20 fmol $mg^{-1}$ cytosol protein. [e]ER and PR < 20 fmol $mg^{-1}$ cytosol protein. [f]For two carcinomas no receptor concentrations were available and for five carcinomas only a negative ER value was obtained.

expressed as fmol $mg^{-1}$ cytosol protein. ER and PR values of smaller than 20 fmol $mg^{-1}$ were considered negative. Carcinomas were classified as hormone receptor positive (at least one receptor concentration positive) or hormone receptor negative (ER-/PR-).

*Tissue preparation*

The fresh tissue samples were snap-frozen in liquid nitrogen and stored at − 70°C until use. Serial cryostat sections were cut at 5 µm, air-dried at least for 2 h and fixed in acetone for 10 min at room temperature (RT). The sections were stored at − 20°C until staining, however, for 4 weeks at maximum.

*Monoclonal antibodies and antisera*

The following MoAbs were used: L 368 against $\beta_2$-microglobulin (Lampson *et al.*, 1983), obtained from Becton-Dickinson, W 6/32 against a monomorphic determinant of HLA-A,B,C antigens (Barnstable *et al.*, 1978), kindly provided by Dr. F. Momburg (DKFZ, Heidelberg, FRG) and L 243 against HLA-DR (Lampson & Levy, 1980), purchased from Becton-Dickinson. Biotinylated horse anti-mouse IgG and avidin-biotinylated peroxidase complex (ABC) were obtained from Vector.

*Immunoperoxidase staining*

The sections were thawed, fixed in acetone for 10 min at RT and rehydrated in PBS (0.04 M, pH 7.4). They were incubated with the first antibody for 30 min at 37°C, then with the second biotinylated horse anti-mouse IgG for 30 min at RT and finally with the ABC for 45 min at RT. After each incubation step the sections were washed three times in PBS. Peroxidase was visualised with DAB in 0.06% hydrogen peroxide. The colour of the DAB precipitation product was intensified by 0.5% copper sulphate in physiological saline. The sections were counterstained with hematoxylin. Negative controls were carried out by replacing the primary antibody by PBS. Stromal cells which were ubiquitously present served as intrinsic positive controls for the immunoreactivity of the MoAbs. Blocking of endogenous peroxidase activity was not necessary since endogenous peroxidase activity, if present at all, did not interfere with the interpretation of the specific staining results.

*Interpretation of immunohistological results*

In order to evaluate the antigen expression in a semiquantitative manner, the following score was applied:

No staining (−); slight staining irrespective of the number of positive tumour (or epithelial) cells, or moderate to strong staining of less than one third of the cells (+); moderate staining of more than one third of the cells, or strong staining of one to two thirds of the cells (+ +); strong staining of more than two thirds of the cells (+ + +). If residual non-neoplastic epithelial structures were found in the carcinoma sections they were evaluated separately.

*Follow-up*

Follow-up data of patients with malignancies were collected by means of inquiries of the treating physicians and by review of the medical records of the Surgical Ambulance, Evangelisches Diakoniekrankenhaus, and the Department of Radiotherapy, University of Freiburg. Patients with distant metastases at the time of diagnosis or whose M-status was unknown were excluded from the follow-up ($n$ = 6). Information was retained about the vital status, recurrence of cancer and the cause of death. The date of histopathological diagnosis of the carcinoma was regarded as the start of follow-up. Data ($n$ = 54) include the date of the last observation of patients alive or the date of death from patients who died from causes other than carcinoma, or from those whose cause of death was unknown. For five patients no information about their postsurgical status could be obtained; they were not considered for the analysis of overall survival. For the analysis of disease-free survival 48 cases provided data. Six patients for whom no information on the relapse was available were excluded. The follow-up time ranged from 26 to 81 months (median 45 months).

*Statistical methods*

The statistical analysis of the data was performed by the Department of Medical Biometrics and Medical Informatics, University of Freiburg. To check for correlations between the expression of MHC antigens and the histological type of lesions and to test the difference in staining results between benign and malignant tumours the $\chi^2$ test was used, while the Sign test was employed to correlate antigen expression in tumor cells with the expression in residual non-neoplastic epithelium. Survival curves were estimated according to Kaplan-Meier and the log-rank, Wilcoxon and the likelihood ratio test were applied as tests of significance. The computing was done by the SAS procedure LIFETEST.

The identification of independent prognostic effects of MHC antigen expression was assessed by the Cox multivariate regression analysis. The characteristics which were included were the pT-stage grouped in two categories, T1–2 and T3–4; two groups formed according to the number of axillary lymph node metastases; and those without, or with up to 3, and those with more than 3 positive nodes. Carcinomas were classified as hormone receptor positive or negative. The age of patients was included as a continuous variable. If any of the above information was not available, patients were excluded, resulting in 63 patients studied for overall and 62 patients for disease-free survival. The Cox regression analysis was computed with BMDP2L, from BMDP Statistical Software, Los Angeles, California. The computing comprised the elimination of single covariates tested against the complete model as well as stepwise-up and stepwise-down regressions. To test for significance the Wald, likelihood ratio, score and $\chi^2$ test were applied.

### Results

*Immunohistology*

The staining results of the benign breast lesions are summarised in Table III. No benign tissue was completely negative for $\beta_2$m or HLA-A,B,C antigens. On comparing anti-$\beta_2$m MoAb with anti-HLA-A,B,C MoAb staining, equal or weaker scores were found for the latter. The antigens were located on the cell membranes as well as in the cytoplasm. Heterogeneity of antigen expression (Figure 1) was observed

**Table III** Staining results of benign breast lesions

| *MoAb* | *Staining score* – | + | + + | + + + | *Total* |
|---|---|---|---|---|---|
| L 368 | 0 | 2 | 14 | 15 | 31 |
| W 6/32 | 0 | 16 | 15 | 0 | 31 |
| L 243 | 3 | 19 | 9 | 0 | 31 |

**Table IV** Staining results of malignant breast lesions

| *MoAb* | *Staining score* – | + | + + | + + + | *Total* |
|---|---|---|---|---|---|
| L 368 | 3 | 26 | 32 | 16 | 77 |
| W 6/32 | 20 | 41 | 13 | 3 | 77 |
| L 243 | 67 | 8 | 2 | 0 | 77 |

**Figure 1** Mastopathy showing heterogeneous expression of HLA-DR. Hematoxylin counterstain. Scale bar: 50 μm.

**Figure 2** Predominantly membranous staining of an invasive ductal carcinoma by anti-$\beta_2$m. Hematoxylin counterstain. Scale bar: 50 μm.

for $\beta_2$m, HLA-A,B,C and HLA-DR. Three tissues (all mastopathies) were negative for HLA-DR. Its expression in the remaining samples generally was lower compared to the expression of $\beta_2$m or HLA-A,B,C. In just one case of hyperplasia and in the gynaecomastia, higher scores were obtained with MoAb L 243.

No correlation was found between the histological type of the benign lesions and the immunohistological demonstration of $\beta_2$m or MHC antigens ($\chi^2$ test). Table IV displays the results obtained with malignant tumours, which showed membrane as well as cytoplasmic staining patterns (Figures 2 and 3). In contrast to benign lesions, the breast carcinomas were characterised by a reduction in the expression of $\beta_2$m and MHC antigens. This difference proved to be statistically significant ($\chi^2$ test, L 368; $P < 0.05$; W 6/32; $P < 0.01$; L 243; $P < 0.001$). Three of 77 malignancies were completely negative for $\beta_2$m and 20/77 for HLA-A,B,C antigens. In comparison to MoAb L 368, MoAb W 6/32 resulted in lower staining scores in carcinomas as well.

By far the majority of malignancies (67/77) proved to be HLA-DR-negative. When carcinomas were positive, the HLA-DR expression was always lower than the expression of HLA-A,B,C or $\beta_2$m, and the staining was situated in tumour areas which were also HLA-A,B,C-positive. No tumour showed staining of all carcinoma cells with MoAb L 243. No correlation was found between the histological type, the expression pTNM stage, the ER or PR level of the carcinomas and the demonstration of MHC antigens. In 13/77 carcinoma samples residual non-neoplastic gland epithelium could be detected (Figure 4). Its antigen expression was evaluated separately and compared to that in the tumour cells (Table V). For MoAbs L 368 and W 6/32 no significant difference in staining between cancer cells and epithelium was found, while, the lower expression of HLA-DR in carcinoma cells was significant.

**Figure 3** Cytoplasmic HLA-A,B,C expression in an invasive lobular carcinoma. Hematoxylin counterstain. Scale bar: 50 μm.

*Survival analysis – Kaplan-Meier estimates*

*Overall survival* Significant differences between survival curves were obtained for the variables: nodal status ($P < 0.01$) and hormone receptor status ($P < 0.01$). Patients with not more than 3 positive lymph nodes and hormone receptor positive tumours were characterised by a better prognosis regarding overall survival. According to the $\beta_2$m

**Figure 4** Residual non-neoplastic epithelium strongly stained for HLA-DR whereas adjacent carcinoma cells are negative. Hematoxylin counterstain. Scale bar: 50 μm.

expression the patients were classified into three groups: − and + ($n = 25$); + + ($n = 26$); + + + ($n = 15$). For HLA-A,B,C the following classes were formed: − ($n = 16$); + ($n = 36$); + + and + + + ($n = 14$). HLA-DR expression resulted in two classes: − ($n = 57$); + and + + ($n = 9$). None of these 3 parameters produced significant differences in the survival curves (Figure 5).

*Disease-free survival* In contrast to overall survival, the pT-stage influenced disease-free survival in our cohort ($P < 0.01$). Axillary lymph node status ($P < 0.01$) and hormone receptor status ($P < 0.05$) also resulted in significantly different disease-free survival curves. Patients with smaller tumours, less than 4 axillary lymph node metastases or hormone receptor positive carcinomas, showed a more favourable prognosis. No effect of $\beta_2$m, HLA-A,B,C or HLA-DR expression on disease-free survival was observed using the same classification of patients as for overall survival (Figure 6).

*Cox analysis* To exclude the theoretical possibility that the effect of antigen expression in the Kaplan-Meier estimates was covered by other factors Cox multivariate regression analyses were carried out. The Cox analysis of overall survival data resulted in only two variables being of prognsotic importance irrespective of the computing method, i.e., the number of axillary lymph nodes ($P < 0.01$) and the hormone receptor status ($P < 0.01$). For the disease-free survival data, no effect of MHC expression was seen. Again, the number of positive axillary lymph nodes ($P < 0.05$) and the hormone receptor status ($P < 0.05$) were of importance, together with the pT-stage, only in the stepwise-up regression ($P < 0.01$).

**Table V** Staining results of carcinoma cells compared to those of residual non-neoplastic epithelium found in the same section

| *MoAb* | *C<E*[a] | *C = E*[b] | *C>E*[c] | *Total* | *P*[d] |
|---|---|---|---|---|---|
| L 368 | 6 | 6 | 1 | 13 | 0.1250 |
| W 6/32 | 5 | 7 | 1 | 13 | 0.2188 |
| L 243 | 10 | 3 | 0 | 13 | 0.0020 |

[a]Carcinoma cells stained weaker than residual epithelium. [b]Carcinoma cells stained like residual epithelium. [c]Carcinoma cells stained stronger than residual epithelium. [d]Level of significance according to the sign test.

### Discussion

The immunohistological examination of 31 benign and 77 malignant human breast tumours revealed a lower expression of HLA-A,B,C and -DR antigens and $\beta_2$m in the malignant tumours. In 13 carcinoma samples which included residual gland epithelium, the HLA-DR expression in the tumour cells was reduced compared to their normal counterparts.

**Figure 5** Kaplan-Meier estimates for overall survival. The classification of the carcinoma patients according to $\beta_2$m (upper graph), HLA-A,B,C (middle graph) and HLA-DR (lower graph) expression did not result in different clinical courses. The solid lines represent carcinomas which were scored as '−' or '+' for $\beta_2$m or '−' for HLA-A,B,C and HLA-DR, respectively. The dashed lines represent carcinomas scored as '+ +' for $\beta_2$m, '+' for HLA-A,B,C and '+' or '+ +' for HLA-DR. The dotted lines represent carcinomas scored as '+ + +' for $\beta_2$m and '+ +' or '+ + +' for HLA-A,B,C. The $P$-values obtained by the logrank, Wilcoxon and likelihood ratio tests were 0.44, 0.32 and 0.53, respectively for $\beta_2$m; 0.38, 0.35 and 0.36, respectively for HLA-A,B,C; 0.48, 0.60 and 0.54, respectively for HLA-DR.

**Figure 6** Kaplan-Meier estimates for disease-free survival. The classification of the carcinoma patients according to $\beta_2$m (upper graph), HLA-A,B,C (middle graph) and HLA-DR (lower graph) expression did not result in different clinical courses. The solid lines represent carcinomas which were scored as −' or '+' for $\beta_2$m or '−' for HLA-A,B,C and HLA-DR, respectively. The dashed lines represent carcinomas scored as '+ +' for $\beta_2$m, '+' for HLA-A,B,C and '+' or '+ +' for HLA-DR. The dotted lines represent carcinomas scored as '+ + + +' for $\beta_2$m and '+ +' or '+ + +' for HLA-A,B,C. The $P$-values obtained by the logrank, Wilcoxon and likelihood ratio tests were 0.33, 0.28 and 0.53, respectively for $\beta_2$m; 0.38, 0.27 and 0.40, respectively for HLA-A,B,C; 0.68, 0.57 and 0.83, respectively for HLA-DR.

The high expression of HLA-A,B,C and $\beta_2$m and a lower expression of HLA-DR in benign lesions are in concordance with the results of other studies (Weiss *et al.*, 1981; Bhan & DesMarais, 1983; Rowe & Beverley, 1984; Sidky & Walker, 1984; Whitwell *et al.*, 1984; Zuk & Walker, 1987) and correspond to the demonstration of these antigens in normal human breast tissue (Daar *et al.*, 1984*a*, 1984*b*; Natali *et al.*, 1983). Likewise, the reduction in expression of MHC antigens and $\beta_2$m in malignant breast tumours has been already described by other authors (Fleming *et al.*, 1981; Natali *et al.*, 1981, 1983, 1984; Bhan & DesMarais, 1983; Rowe & Beverley, 1984; Sawtell *et al.*, 1984; Sidky & Walker, 1984; Whitwell *et al.*, 1984; Göttlinger *et al.*, 1985; Hurlimann & Saraga, 1985; Perez *et al.*, 1986; Zuk & Walker, 1987), however the positivity rates of the carcinomas in these studies vary considerably. The different MoAbs and methods applied, other criteria for interpretation of staining results and the heterogeneous patterns of antigen expression might explain the divergent results.

The expression, or absence, of MHC antigens in breast carcinomas did not apparently influence the overall, or disease-free, survival. The structure of our cohort for age and tumour features is comparable to that of the studies by Shek & Godolphin (1988) and Alexieva-Figusch *et al.* (1988), who described the prognostic relevance of the classic parameters such as tumour size, axillary lymph node status, ER and PR levels. This comparability weakens the possible explanation of our results being merely an outcome of a selective collection of patients. In addition, our follow-up period is almost identical to that of Alexieva-Figusch *et al.*, exact data are not given by Shek & Godolphin. The reliability of our results is further strengthened by the identification of the axillary lymph node and hormone receptor status as independent prognostic factors for overall and disease-free survival. These parameters confirm the observations of the studies cited above as well as those from other investigations (Nemoto *et al.*, 1980; Baak *et al.*, 1985; Bryan *et al.*, 1986; Fisher *et al.*, 1987; Russo *et al.*, 1987; Todd *et al.*, 1987; Chevallier *et al.*, 1988).

In colorectal, but not in breast, carcinomas Stein *et al.* (1988) could not find any influence of MHC antigens on survival. Interestingly, this group described an inverse correlation between the expression of HLA-A,B,C antigens and the degree of differentiation in these carcinomas (Momburg *et al.*, 1986). Poorly differentiated carcinomas are characterised by a worse clinical prognosis (Morson & Dawson, 1979). Thus, it seems that a similar discrepancy may also exist for breast carcinomas. The correlation between the reduction in MHC class I antigen expression and poor tumour differentiation, a bad prognostic feature by itself, favours the assumption that loss of MHC class I antigen may impair prognosis. However, this idea is not confirmed by survival analysis.

Several other aspects have to be considered before a prognostic relevance of MHC antigens in breast carcinomas can be excluded. From our study the possibility cannot be ruled out that immunological mechanisms may be important in the regulation of tumour growth of carcinoma subgroups, e.g., carcinomas of small size. Vánky *et al.* (1983*b*) and Klein (1988) reported that, in an *in vitro* assay, tumour cells from patients without metastases were lysed by autologous peripheral blood lymphocytes but not by cells of patients with metastases, and this antitumour reactivity correlated with the survival of the tumour patients (Vánky *et al.*, 1983*a*,*b*). In melanoma, Bröcker *et al.* (1985) observed shorter disease-free intervals in the case of patients with stage I tumours positive for HLA-DR, while Fossati *et al.* (1986) could not detect a correlation between the presence of HLA-DR and survival in stage II disease. *In vitro* tests seem to indicate that MHC class I antigen expression on tumour cells is more relevant for putative *in vivo* tumour cytotoxicity by T-cells than class II antigens. The differential expression of MHC class I antigens has recently been described in colorectal carcinomas (Rees *et al.*, 1988; Smith *et al.*, 1988; López-Nevot *et al.*, 1989; Momburg *et al.*, 1989), gastric and laryngeal carcinomas (López-Nevot *et al.*, 1989) and breast carcinomas (Müller & Stutte, 1988). An impact of MHC class I subtypes on the clinical course of human breast carcinomas is suggested by the latter group, who demonstrated that HLA-$A_2$ expression in breast carcinomas is conversely related to the number of axillary lymph node metastases. When the same tumours were stained with a MoAb specific for HLA-A,B such a correlation was not found. From these results it was concluded that HLA-B expression reduced the immunogenicity of breast carcinomas which resulted in a higher frequency of metastases. Similar results concerning the MHC class I antigens were reported by Eisenbach *et al.* (1983, 1984, 1985) based on a mouse tumour model: the imbalance of H-2K/H-2D in favour of H-2D enhanced the metastatic capacity of the tumour cells.

In appraising the relevance of MHC class II expression on tumour cells, it is interesting to note that these antigens have also been correlated with suppressive effects (Parmiani *et al.*, 1985). Although there is little information on the genetics of suppression in man, suppressive phenomena seem to be associated with HLA-DQ (Oliveira & Mitchinson, 1989). For 6 breast carcinomas the differential expression of MHC class II antigens has been described by Natali *et al.* (1986). In patients with metastatic melanoma the same authors observed a more favourable prognosis when the metastases expressed HLA-DQ, which does not fit with the association of HLA-DQ and a supposed suppression of immune response as mentioned above.

The experimental results providing strong evidence for the importance of MHC antigen expression on tumour cells for an effective antitumour immune defence against tumour cell growth are not confirmed by our analysis of tumour patients survival data. However, for breast carcinoma patients' surveillance, our follow-up period is relatively short, since recurrences may appear even after 20 years. In addition, the percentage of data for the Kaplan-Meier estimates was rather high (around 70%). The identification of nodal and hormone receptor status as prognostic parameters in our cohort and the comparison of our median follow-up period with other studies proved that this interval was, nevertheless, long enough for reliable interpretations. However, the study of larger numbers of carcinoma patients and a longer follow-up period are necessary to draw definitive conclusions.

The technical help from M. Polack and F. Jehle is highly appreciated. We thank Professor Slanina, Department of Radiotherapy, University of Freiburg and Professor Gropp, Head of the Department of Surgery, Evangelisches Diakoniekrankenhaus, Freiburg for their permission to collect follow-up data from the patients' files. In addition, we are indebted to Professor Gropp for supplying breast tissue specimens. This work was supported by 'Deutsche Krebshilfe e.V., Dr Mildred-Scheel Stiftung für Krebsforschung'.

### References

ANICHINI, A., FOSSATI, G. & PARMIANI, G. (1985). Clonal analysis of cytotoxic T-lymphocyte response to autologous human metastatic melanoma. *Int. J. Cancer*, **35**, 683.

ALEXIEVA-FIGUSCH, J., VAN PUTTEN, W.L.J., BLANKENSTEIN, M.A., BLONK-VAN DER WIJST, J. & KLIJN, J.G.M. (1988). The prognostic value and relationships of patients characteristics, estrogen and progestin receptors, and site of relapse in primary breast cancer. *Cancer*, **61**, 758.

BAAK, N.P.A., VAN DOP, H., KURVER, P.H.J. & HERMANS, J. (1985). The value of morphometry to classic prognosticators in breast cancer. *Cancer*, **56**, 374.

BARNSTABLE, C.J., BODMER, W.F., BROWN, G. & 4 others (1978). Production of monoclonal antibodies to group A erythrocytes, HLA and other human cell surface antigens – new tools for genetic analysis. *Cell*, **14**, 9.

BHAN, A.K. & DESMARAIS, C.L. (1983). Immunohistologic characterization of major histocompatibility antigens and inflammatory cellular infiltrate in human breast cancer. *J. Natl Cancer Inst.*, **71**, 507.

BLOOM, H.J.G. & RICHARDSON, W.W. (1957). Histological grading and prognosis in breast cancer. *Br. J. Cancer.*, **11**, 359.

BRÖCKER, E.B., SUTER, L., BRÜGGEN, J., RUITER, D., MACHER, E. & SORG, C. (1985). Phenotypic dynamics of tumor progression in human malignant melanoma. *Int. J. Cancer*, **36**, 29,

BRYAN, R.M., MERCER, R.J., BENNETT, R.C. & RENNIE, G.C. (1986). Prognostic factors in breast cancer and the development of a prognostic index. *Br. J. Surg.*, **73**, 267.

CHEVALLIER, B., HEINTZMANN, F., MOSSERI, V. & 7 others (1988). Prognostic value of estrogen and progesterone receptors in operable breast cancer. *Cancer*, **62**, 2517.

DAAR, A.S., FUGGLE, S.V., FABRE, J.W., TING, A. & MORRIS, P.J. (1984*a*). The detailed distribution of HLA-A,B,C antigens in normal human organs. *Transplantation*, **38**, 287.

DAAR, A.S., FUGGLE, S.V., FABRE, J.W., TING, A. & MORRIS, P.J. (1984*b*). The detailed distribution of MHC class II antigens in normal human organs. *Transplantation*, **38**, 293.

DARROW, T.L., SLINGLUFF, C.L. & SEIGLER, H.F. (1989). The role of HLA class I antigens in recognition of melanoma cells by tumor-specific cytotoxic T lymphocytes. *J. Immunol.*, **142**, 3329.

DI BELLO, M., LUCCHINI, V., CHIARI, S. & 4 others (1988). DR antigen expression on ovarian carcinoma cells does not correlate with their capacity to elicit an autologous proliferative response. *Cancer Immunol. Immunother.*, **27**, 63.

DOHERTY, P.C., KNOWLES, B.B. & WETTSTEIN, P.J. (1984). Immunological surveillance of tumors in the context of major histocompatibility complex restriction of T cell function. *Adv. Cancer Res.*, **42**, 1.

EISENBACH, L., SEGAL, S. & FELDMAN, M. (1983). MHC imbalance and metastatic spread in Lewis lung carcinoma clones. *Int. J. Cancer*, **32**, 113.

EISENBACH, L., HOLLANDER, N., GREENFELD, L., YAKOR, H., SEGAL, S. & FELDMAN, M. (1984). The differential expression of H-2K versus H-2D antigens, distinguishing high-metastatic from low-metastatic clones, is correlated with the immunogenic properties of the tumor cells. *Int. J. Cancer*, **34**, 567.

EISENBACH, L., HOLLANDER, N., SEGAL, S. & FELDMAN, M. (1985). The differential expression of class I major histocompatibility complex antigens controls the metastatic properties of tumor cells. *Transplant. Proc.*, **17**, 729.

FISHER, E.R., SASS, R. & FISHER, B. (1987). Pathologic findings from the national surgical adjuvant breast project. Correlations with concordant and discordant estrogen and progesterone receptors. *Cancer*, **59**, 1554.

FLEMING, K.A., MCMICHAEL, A., MORTON, J.A., WOODS, J. & MCGEE, J.O.D. (1981). Distribution of HLA class I antigens in normal human tissue and in mammary cancer. *J. Clin. Pathol.*, **34**, 779.

FOSSATI, G., TARAMELLI, D., BALSARI, A., BOGDANOVICH, G., ANDREOLA, S. & PARMIANI, G. (1984). Primary but not metastatic human melanomas expressing DR antigens stimulate autologous lymphocytes. *Int. J. Cancer*, **33**, 591.

FOSSATI, G., ANICHINI, A., TARAMELLI, D. & 4 others (1986). Immune response to autologous human melanoma: implication of class I and II MHC products. *Biochim. Biophys. Acta*, **865**, 235.

FREEDMAN, L.S., EDWARDS, D.N., MCCONNELL, E.M. & DOWNHAM, D.Y. (1979). Histological grade and other prognostic factors in relation to survival of patients with breast cancer. *Br. J. Cancer*, **40**, 44.

GÖTTLINGER, H.G., RIEBER, P., GOKEL, J.M., LOHE, K.J. & RIETHMÜLLER, G. (1985). Infiltrating mononuclear cells in human breast carcinoma: predominance of T4 + monocytic cells in the tumor stroma. *Int. J. Cancer*, **35**, 199.

GUERRY, D., ALEXANDER, M.A., HERLYN, M.F. & 4 others (1984). HLA-DR histocompatibility leukocyte antigens permit cultured human melanoma cells from early but not advanced disease to stimulate autologous lymphocytes. *J. Clin. Invest.*, **73**, 267.

GUILLEMOT, F., AUFFRAY, C., ORR, H.T. & STROMINGER, J.L. (1988). MHC antigen genes. In: B.D. Hames, D.M. Glover (eds), *Molecular Immunology*, p. 81, IRL Press: Oxford.

HÄMMERLING, G.J., KLAR, D., PÜLM, W., MOMBURG, F. & MOLDENHAUER, G. (1987). The influence of major histocompatibility complex class I antigens on tumor growth and metastasis. *Biochim. Biophys. Acta*, **907**, 245.

HAYBITTLE, J.L., BLAMEY, R.W., ELSTON, C.W. & 5 others (1982). A prognostic index in primary breast cancer. *Br. J. Cancer*, **45**, 361.

HEDRICK, S.M. (1988). Specifity of the T cell receptor for antigen. *Adv. Immunol.*, **43**, 193.

HURLIMANN, J. & SARAGA, P. (1985). Mononuclear cells infiltrating human mammary carcinomas: immunohistochemical analysis with monoclonal antibodies. *Int. J. Cancer*, **35**, 753.

ITOH, K., PLATSOUCAS, C.D. & BALCH, C.M. (1988). Autologous tumor-specific cytoxic T lymphocytes in the infiltrate of human metastatic melanomas. *J. Exp. Med.*, **168**, 1419.

KLEIN, E. (1988). Interaction of T lymphocytes with 1) EBV carrying B cells, 2) with carcinoma cells. *16th International I.S.O.B.M. Congress:* Barcelona, Spain, p. 60.

KNUTH, A., WÖLFEL, T., KLEHMANN, E., BOON, T. & MEYER ZUM BÜSCHENFELDE, K.H. (1989). Cytolytic T-cell clones against an autologous human melanoma: specificity study and definition of three antigens by immunoselection. *Proc. Natl Acad. Sci.*, **86**, 2804.

LAMPSON, L.A. & LEVY, R. (1980). Two populations of Ia-like molecules on a human B cell line. *J. Immunol.*, **125**, 293.

LAMPSON, L.A., FISHER, C.A. & WHELAN, J.P. (1983). Striking paucity of HLA-A,B,C and $\beta_2$-microglobulin on human neuroblastoma cell lines. *J. Immunol.*, **130**, 2471.

LÓPEZ-NEVOT, M.A., ESTEBAN, F., FERRÓN, A. & 6 others (1989). HLA class I gene expression on human primary tumours and autologous metastases: demonstration of selective losses of HLA antigens on colorectal, gastric and laryngeal carcinomas. *Br. J. Cancer*, **59**, 221.

MOMBURG, F., DEGENER, T., BACCHUS, E., MOLDENHAUER, G., HÄMMERLING, G.J. & MÖLLER, P. (1986). Loss of HLA-A,B,C and *de novo* expression of HLA-D in colorectal cancer. *Int. J. Cancer*, **37**, 179.

MOMBURG, F., ZIEGLER, A., HARPRECHT, J., MÖLLER, P., MOLDENHAUER, G. & HÄMMERLING, G.J. (1989). Selective loss of HLA-A or HLA-B antigen expression in colon carcinoma. *J. Immunol.*, **142**, 352.

MORSON, B.C. & DAWSON, I.M.P. (1979). *Gastrointestinal Pathology*, 2nd ed., p. 649, Blackwell: Oxford.

MÜLLER, H. & STUTTE, H.J. (1988). The correlation of major histocompatibility complex gene product expression with proliferative activity and metastatic competence in ductal carcinoma of the breast. *Verh. Dtsch. Ges. Path.*, **72**, 260.

NATALI, P.G., DE MARTINO, C., QUARANTA, V., BIGOTTI, A. & PELLEGRINO, M.A. (1981). Changes in Ia-like antigen expression on malignant human cells. *Immunogenetics*, **12**, 409.

NATALI, P.G., GIACOMINI, P., BIGOTTI, A., IMAI, K., NG, A.K. & FERRONE, S. (1983). Heterogeneity in the expression of HLA and tumor-associated antigens by surgically removed and cultured breast carcinoma cells. *Cancer Res.*, **43**, 660.

NATALI, P.G., BIGOTTI, A., NICOTRA, M.R., VIORA, M., MANFREDI, D. & FERRONE, S. (1984). Distribution of human class I (HLA-A,B,C) histocompatibility antigens in normal and malignant tissues of nonlymphoid origin. *Cancer Res.*, **44**, 4679.

NATALI, P.G., BIGOTTI, A., CAVALIERI, R. & 6 others (1986). Gene products of the HLA-D region in normal and malignant tissues of nonlymphoid origin. *Hum. Immunol.*, **15**, 220.

NEMOTO, T., VAN, J., BEDWANI, R.N., BAKER, H.W., MCGREGOR, F.H. & MURPHY, G.P. (1980). Management and survival of female breast cancer: results of a national survey by the American college of surgeons. *Cancer*, **45**, 2917.

OLIVEIRA, D.B.G. & MITCHINSON, N.A. (1989). Immune suppression genes. *Clin. Exp. Immunol*, **75**, 166.

PARMIANI, G., FOSSATI, G., TARAMELLI, D. & 5 others (1985). Autologous cellular immune response to primary and metastatic human melanomas and its regulation by DR antigens expressed on tumor cells. *Cancer Metast. Rev.*, **4**, 7.

PÉREZ, M., CABRERA, T., LOPEZ-NEVOT, M.A. & 4 others (1986). Heterogeneity of the expression of class I and II HLA antigens in human breast carcinoma. *J. Immunogen.*, **13**, 247.

REES, R.C., BUCKLE, A.M., GELSTHORPE, K. & 4 others (1988). Loss of polymorphic A and B locus HLA antigens in colon carcinoma. *Br. J. Cancer*, **57**, 374.

REINER, A., HOLZNER, J.H., REINER, G. & 4 others (1985). Histological grading and prognosis in breast cancer. *Verh. Dtsch. Ges. Path.*, **69**, 365.

ROBERTS, T.E., SHIPTON, U. & MOORE, M. (1987). Role of MHC class-I antigens and the CD3 complex in the lysis of autologous human tumours by T-cell clones. *Int. J. Cancer*, **39**, 436.

ROWE, D.J. & BEVERLEY, P.C.L. (1984). Characterisation of breast cancer infiltrates using monoclonal antibodies to human leucocyte antigens. *Br. J. Cancer*, **49**, 149.

RUSSO, J., FEDERICK, J., OWNBY, H.E. & 5 others (1987). Predictors of recurrence and survival of patients with breast cancer. *Amer. J. Clin. Pathol.*, **88**, 123.

SAWTELL, N.M., DIPERSIO, L., MICHAEL, J.G., PESCE, A.J. & WEISS, M.A. (1984). Expression of normal and tumor-associated antigens in human breast carcinoma. *Lab. Invest.*, **51**, 225.

SHEK, L.L. & GODOLPHIN, W. (1988). Model for breast cancer survival: relative prognostic roles of axillary nodal status, TNM stage, estrogen receptor concentration, and tumor necrosis. *Cancer Res.*, **48**, 5565.

SIDKY, K. & WALKER, R.A. (1984). $\beta_2$-microglobulin in non-malignant and malignant human breast: a feature of differentiation. *J. Pathol.*, **142**, 135.

SMITH, M.E.F., BODMER, W.F. & BODMER, J.G. (1988). Selective loss of HLA-A,B,C locus products in colorectal adenocarcinoma. *Lancet*, **i**, 823.

STEIN, B., MOMBURG, F., SCHWARZ, V., SCHLAG, P., MOLDENHAUER, G. & MÖLLER, P. (1988). Reduction or loss of HLA-A,B,C antigens in colorectal carcinoma appears not to influence survival. *Br. J. Cancer*, **57**, 364.

TANAKA, K., YOSHIOKA, T., BIEBERICH, C. & JAY, G. (1988). Role of the major histocompatibility complex class I antigens in tumor growth and metastasis. *Ann. Rev. Immunol.*, **6**, 359.

TODD, J.H., DOWLE, C., WILLIAMS, M.R. & 5 others (1987). Confirmation of a prognostic index in primary breast cancer. *Br. J. Cancer*, **56**, 489.

UICC (HERMANEK, P., SCHEIBE, O., SPIESSL, B. & WAGNER, G. (eds)) (1987). *TNM-Klassifikation maligner Tumoren*, 4th ed., Springer: Berlin.

VÁNKY, F. (1986). Membrane structures involved in auto-tumor recognition. *Biochim. Biophys. Acta*, **865**, 253.

VÁNKY, F., WILLEMS, J., KREICBERGS, A. & 6 others (1983*a*). Correlation between lymphocyte-mediated auto-tumor reactivities and clinical course. I. Evaluation of 46 patients with sarcoma. *Cancer Immunol. Immunother.*, **16**, 11.

VÁNKY, F., PÉTERFFY, A., BÖÖK, K., WILLEMS, J., KLEIN, E. & KLEIN, G. (1983*b*). Correlation between lymphocyte-mediated anti-tumor reactivities and the clinical course. II. Evaluation of 69 patients with lung carcinoma. *Cancer Immunol. Immunother.*, **16**, 17.

VÁNKY, F., KLEIN, E. & WILLEMS, J. (1985). DR antigens expressed on tumor cells do not contribute to the blastogenetic response of autologous T cells. *Cancer Immunol. Immunother.*, **19**, 219.

VÁNKY, F., ROBERTS, T., KLEIN, E. & WILLEMS, J. (1987). Autotumour immunity in patients with solid tumors: participation of CD3 complex and MHC class I antigens in the lytic interaction. *Immunol. Lett.*, **16**, 21.

WEISS, M.A., MICHAEL, J.G., PESCE, A.J. & DIPERSIO, L. (1981). Heterogeneity of $\beta_2$-microglobulin in human breast carcinoma. *Lab. Invest.*, **45**, 46.

WHITWELL, H.L., HUGHES, H.P.A., MOORE, M. & AHMED, A. (1984). Expression of major histocompatibility antigens and leucocyte infiltration in benign and malignant human breast disease. *Br. J. Cancer*, **49**, 161.

ZUK, J.A. & WALKER, R.A. (1987). Immunohistochemical analysis of HLA antigens and mononuclear infiltrates of benign and malignant breast. *J. Pathol.*, **152**, 275.